# No Ethics Needed? Towards an Ethics of Care for Public and Patient Involvement and Engagement

**DOI:** 10.1111/hex.70638

**Published:** 2026-03-20

**Authors:** Kanwal Mand, Anna Lavis, Melissa Stepney, Roisin Mooney, Kam Bhui

**Affiliations:** ^1^ Department of Applied Health Sciences University of Birmingham Birmingham UK; ^2^ NIHR Oxford Health Biomedical Research Centre Oxford UK; ^3^ Institute for Mental Health University of Birmingham Birmingham UK; ^4^ Department of Psychiatry University of Oxford Oxford UK; ^5^ World Psychiatric Association Collaborating Centre Oxford UK; ^6^ Department of Psychiatry and Nuffield Department of Primary Care Health Sciences Oxford UK; ^7^ Wadham College University of Oxford Oxford UK; ^8^ Centre for Psychiatry and Mental Health Queen Mary University of London London UK

## Abstract

**Introduction:**

Public and Patient Involvement and Engagement (PPIE) enhances the inclusivity, relevance and responsiveness of health research for communities. Although public and patient involvement is grounded in lived experience and typically falls outside formal ethical review, ethical considerations nonetheless arise. This paper therefore examines PPIE concepts and practices through an EoC lens.

**Method:**

Through a thematic, iterative review of the literature, this paper examines PPIE practices, analysing these by drawing on feminist scholarship, notably the EoC perspective and intersectionality, to explore how lived experience is constructed within health research.

**Results:**

The EoC perspective can play an important guide for helping researchers to promote inclusive team cultures, strengthen their PPIE activity and respond to marginalised groups of people in complex situations. It can help to address criticisms of PPIE, on account of the ‘harm’ patient/public contributors can experience based on (hidden) emotional labour, alongside power imbalances, and existent health vulnerabilities. Whilst there exists some guidance concerning PPIE in terms of reporting, the paper proposes an EoC lens underlining five overlapping aspects: attentiveness, responsibility, competence, responsiveness and solidarity to improve inclusivity, mitigate harms and reshape relationships between researchers and participants.

**Conclusion:**

An EoC approach reframes ethics, for PPIE, not as a procedural task. It highlights that an EoC is a form of relational ethics and underlines the ongoing relational practice that makes up PPIE, which demands attentiveness to lived experience, structural inequalities and the emotional realities of involvement. This perspective strengthens the ethical integrity of PPIE by encouraging more reflexive, compassionate and equitable engagement across research contexts.

**Patient or Public Contribution:**

Members of a parent carer network were involved in preparing the manuscript. They reviewed the paper in both a lay summary version and the longer full version, providing written and verbal feedback. Their suggestions have been incorporated into the main text, including the addition of examples in the table.

## 1 Introduction and Context

Public and Patient Involvement and Engagement (PPIE) activities are essential for ensuring that health research, and the intervention and prevention strategies built upon it, are relevant and appropriate for patients and members of the public. In the United Kingdom, PPIE is advocated for by several research and funding organisations such as the Medical Research Council (MRC), Wellcome Trust and the National Institute of Health Research (NIHR). This paper takes, as a starting point, the NIHR's mandate regarding PPIE for addressing health inequalities and empowering individuals and communities through research inclusion [1, 2]. For the NIHR, ‘public and patient involvement in identifying research priorities and designing research’ has—since 2006—emphasised ‘participation from underserved groups’ [2]. As such, the NIHR's strategy for research inclusion emphasises a commitment to equality and diversity given persistent and resistant health disparities as well as an established lack of representativeness in PPIE [2] and in research more generally. This reflects a broader shift towards a research approach that works with and alongside communities rather than on and about them [1]. This bottom‐up approach, based on people and communities ‘lived experience’, aims to address health inequalities that disproportionately affect marginalised groups and those who are underrepresented in research [3].

PPIE participants are usually viewed not as research subjects but as partners in designing and conducting research. PPIE seeks being research inclusive and aims to improve research relevance considering factors such as language and accessibility to address epistemic injustices [4]. Alongside further guidance on research inclusion, others have emphasised the need for conceptual clarity regarding the purposes of PPIE and the roles of ‘lived experience’ researchers, as these are enacted in various ways [5, 6, 7, 8]. For example, patient/public contributors in PPIE bring lived experience; they also act as advisors, answering and improving questionnaires, participating in focus groups or being involved in guiding clinical trials, as well as offering interpretations of the study findings. PPIE members can also act as peer researchers embedded within the research process through approaches like co‐production. PPIE can occur in clinical and non‐clinical settings, within institutions or in the public sphere. PPIE is often used to highlight the importance of engagement, both in disseminating research findings and in participating in research itself [1, 9, 10].

A key tenet of our concern is that PPIE contributors are not viewed in the same manner way as individuals or groups' participation in research for which REC ethical approval is necessary. The NHS Health Research Authority (HRA) offers an online tool to determine whether ethical approval is deemed necessary when participants are involved as research participants from whom data are being collected. PPIE participants are not involved in data generation, but in varying ways are so through the design or shape of research and likely result in an output [11]. PPIE participants form a category of contributors on account of their ‘lived experiences’. ‘Lived experience’ is central for public involvement in research as NIHR guidance suggests, and considers diversity, expertise and relevancy to be significant [10]. What remains obscured, nonetheless, is an exploration of ‘lived experiences’ are produced and expressed and the relationship to knowledge production. The relationship between ‘lived experiences’, knowledge production and social change cannot be considered as a given rational linear process. A focus on process highlights the challenge when ‘lived experience’ is a catch all notion, and critics suggest that ‘incorporating lived experience expertise into dominant academic paradigms [de facto] will not create systemic change’ [7]. The types of experience and contexts of PPIE warrant more careful exploration before and during research studies, considering too that this might be perceived as excessive disclosure or reinforcing stigma and epistemic bias.

This paper proposes that an EoC perspective allows for a critical examination of how categories of ‘lived experience’ necessitate care and being reflexive. The perspective outlines intersecting and overlapping aspects of care that mingle or co‐exist in varieties of categorised experience; these carry power in constituting and shaping expressed ‘lived experiences’. PPIE involves positioned actors operating within hierarchies and power relations, comprising researchers and institutions, which can seem obstructive or oppressive or rule inflexible. PPIE is a space where contestations could be held between and within researchers and experts by experience. An EoC perspective offers a valuable lens through which to examine these dynamics. It highlights ethics not simply as procedural, but as a moral obligation to think deeply and consider the positionality and complexity of ‘lived experiences’, and to provide tailored care and support to derive a more nuanced practice of PPIE. In the context of addressing health inequalities, an EoC approach shifts the emphasis from processes and outcomes to the relational practices that are central to PPIE.

## 2 No Need for Ethics

Although PPIE in research is often deemed to not require formal ethics committee approvals, the NIHR provides resources and guidance, stating that as a ‘guiding rule … work or activity in… [involvement, engagement, participation ‐ PPIE] should “do no harm” to others. On the contrary, it should complement and strengthen them’ [1, p. 10]. The exemption of PPIE activities from formalised ethical scrutiny under the guidance of ‘do no harm’ harks back to a traditional approach that emphasises duty‐based categorical imperatives that hold universal applicability [12].

‘Do no harm’ suggests being cautious of the impact of PPIE activities, and there exist guidance and frameworks for researchers about patient public involvement. For example, the Public Involvement Impact Assessment Framework [13], instigated by the MRC and NIHR, on account of the growth in public involvement, is ‘to assist researchers to design a plan to assess PI [public involvement] impact that will meet the needs of their specific project’ [14, p. 951]. Additionally, the Guidance for Reporting Involvement of Patients and Public (GRIPP) suggests approaches for researchers to report public involvement [15]. Guidelines and frameworks construct public and patient contributors involved in PPIE activities as a rationalised activity made up of processes and procedures, that are collectively followed and regulated, that is, safeguarding policies. This relegates the emotional dimensions of ‘lived experiences’ for both participants and researchers to the margins.

Demarcating ethics as only a matter for data collection and not for PPIE has ‘unintended consequences’ and ‘invisible impacts’ [11]. Amongst health researchers, there is a recognition of a ‘grey area’ concerning the ethical dimensions of PPIE activities that includes administrative aspects involving researchers and patient and public partners [16, 17]. PPIE activities involve (hidden) emotional labour for partners, facilitators and researchers [18, 19]. Whilst PPIE is embedded within institutional structures intended to manage risk and safeguard participant well‐being, ethical issues still arise. These include concerns about tokenistic involvement, and the labour expected from patient partners as they contribute and when these are not addressed [19]. These, to date, largely underexamined dimensions of PPIE have led to calls for ethical guidance in PPIE as a ‘strategic priority’ [17, p. 7]. In response to that call, what follows is a proposal to utilise an EoC perspective when undertaking PPIE. This provides a more responsive alternative to formal ethical processes, one that emphasises the consequences of involvement as a relational responsibility and considers interdependence, as well as an attentiveness to the lived realities of those involved.

## 3 An Ethics of Care Perspective for PPIE

An EoC departs from traditional approaches to ethics in that it underlines ethics as being based on a responsibility of ‘care’ that is not abstract and universal (deontological), or utilitarian (based on logic and consequence) [12]. The EoC has its roots in a feminist approach and is underpinned by a moral responsibility ‘to care’, with the focus placed on relationships and interdependencies, as opposed to ethical processes that are technocratic and abstract. It was first introduced by psychologist Gilligan [20], whose research found that young girls' moral reasoning, in contrast to boys', tended to approach ethical issues through a lens of care and compassion rather than through principles of justice and autonomy. Gilligan [20] argued for a reconceptualisation of ethics by prioritising relationships, and Tronto [21] furthered this by incorporating a socio‐political perspective, notably in response to systemic injustice and inequality. Tronto [21] posits care as praxis approached through five interrelated and overlapping aspects: (A) caring for (attentiveness to need), (B) caring about (moral responsibility to act), (C) caregiving (competent capacity), (D) care receiving (responsiveness to how care is experienced) and (E) caring with (solidarity based on mutual respect and equity) [20, 21]. Ethics are considered part of the relational dynamics of PPIE that require care.

Table 1 summarises EoC in terms of practice and its translation to PPIE.

**Table 1:** EoC practices and their applicability to PPIE.

EoC involves (5 aspects)	Practice	Example health research PPIE
Caring for *Attentiveness*‐Awareness	Caring that notes the need to be inclusive and to address barriers.	A diabetes study offers online sessions and phone options for rural participants who cannot travel.
Caring about *Responsibility*	Taking care of, and assuming responsibility to care (about how to gather knowledge).	Researchers create safe spaces with support contacts for participants.
Caregiving *Competence*	Researchers have competence and resources for caregiving.	A cancer research team trains staff in cultural competence and trauma‐informed communication.
	Reflect on practice.	
Care receiving *Responsiveness*	How care receivers respond to the care they receive.	Stroke rehabilitation researchers rewrite materials in plain language after participants say jargon made them feel excluded.
	Being aware of the impact on participants.	
Caring with *Solidarity*	Creating alliances, working together to fight inequalities.	Maternal health researchers partner with community advocates to co‐design solutions and challenge barriers like lack of translation services.

As a perspective, the EoC has informed nursing and medical practitioners as ‘professionals’ who ‘make ethical decisions that uphold effective care processes during complex and unpredictable health scenarios’ [22, p. 89]. In health research, not PPIE, an EoC perspective is adopted specifically involving participatory research practices [23, 24, 25]. While EoC is not explicitly used, the argument for ethical guidance in PPIE, as noted above, echoes core principles of EoC, particularly a call for reflexivity on power relations, trust and responsibility [17]. An exception is an Australian study focusing on women's health care experiences following pregnancy [26]. Known locally as Consumer and Community Involvement (CCI), this project is distinctive as it explicitly incorporates an EoC perspective with for PPIE participants identified as ‘Lived Experience Expertise’ group members (LEE). The adoption of five interrelated ‘care’ perspectives with LEE members for PPIE addresses concerns about involvement and power imbalances between researchers and health consumers (LEE members) [26, p. 7].

NIHR data show that PPIE participants are predominantly white, female, over 50 and heterosexual [27]. Variations exist, for example, Mah et al. [28] found ‘PPI advisors were often older, of White ethnicity, highly educated with professional backgrounds’ [28, p. 4]. Meanwhile, comparator data (NIHR 2018–2019) show children and young people (CYP) from minority communities are underrepresented, and this also ties into specific geographical localities [27]. Making research relevant through PPIE involvement is important as ‘people who are most likely to experience poor outcomes are also those *least* likely to take part in conversations about research, attend research‐related events, join advisory groups or enrol as research participants’ [29, p. 3, emphasis mine]. Being underrepresented in PPIE arguably has profound consequences for minority communities, given that they experience long‐standing health inequalities and multiple barriers in accessing healthcare services [6, 30]. People from marginalised communities are unlikely to participate in PPIE activities due to the power relations in the context of rationalised structures of PPIE processes and procedures [6, 31].

Against this background, an EoC perspective underlines the need to ‘care about’ the underrepresentation of participants from marginal and minority communities, especially emphasising inclusivity and participation based on ‘lived experiences’ as a bedrock of PPIE. Here, EoC takes on a research inclusion lens. An EoC perspective proposes a moral obligation to care for and about disparities in participation within historical and current power structures and social inequalities. It emphasises the ethical responsibility of researchers to understand how participation impacts the practice of PPIE and vice versa. From an EoC perspective, PPIE involves being reflexive about knowledge production away from a ‘neutral’ method and instrument for generating outcomes [25], towards ‘a principled framework within which to practise research’ so as not to reproduce, reinforce, perpetuate inequity through PPIE [32, p. 216].

In PPIE for health‐related research, there is an emphasis on the ‘lived experience’ of participants made up of current or recovering patients, the public, carers (including children), (peer) researchers and PPIE facilitators. In the United Kingdom, the emphasis on ‘lived experience’ in PPIE traces back to the 1983 Griffiths Report. The report highlighted the need to ascertain ‘how well a service is being delivered at the local level by obtaining the experience and perceptions of patients and the community’ [33, Patients and Community section]. At that time, PPIE was supported through Community Health Councils (CHCs) who represented the public with regards to health services. Although CHCs were subsequently abolished, legislation introduced in 2003 required the NHS to involve patients in service provision. Today, ‘lived experience’ is fundamental to PPIE. Listening to and taking account of ‘lived experiences’ is considered central in addressing epistemic injustice and health inequalities, especially for underrepresented communities, and in shaping research design, delivery and research relevance [1]. ‘Lived experiences’ are also made up of biases, discrimination, judgements and stereotypes, which can be enacted in PPIE settings, and further existing inequalities and epistemic injustices. Increasingly, methods involving co‐production are seen as ways to empower agency and create knowledge grounded in ‘lived experience’ [1, 34]. There is, nevertheless, a need to critically consider the concept of ‘lived experience’, how it relates to the categorisation of participation in research and for further exploration of the structures within which PPIE to address power imbalances and health inequalities. A focus on ‘lived experiences’ in PPIE should include the possibilities for public, patient partners to voice their experiences of services and of health conditions that otherwise is unaddressed. PPIE participants' ‘lived experience’ of health services is inherently subjective and innately informed by emotions for participants [35]. From an EoC standpoint, PPIE participants' emotional and embodied experiences must be considered alongside their existing socio‐economic, political and health conditions. Caring for and about ‘lived experiences’ requires taking responsibility for participants who often face their own vulnerabilities while engaging in PPIE. It is important to recognise the care burden placed on participants, as it emerges within power relations during and after PPIE activities, and in the dynamics between participants, PPIE facilitators and researchers. The institutional contexts in which PPIE takes place are shaped by ‘feeling rules’ that structure interactions and, by extension, influence knowledge production. For example, PPIE meetings between researchers and participants contain norms of what is appropriate to feel, reveal and display [35]; see also [36].

To tackle health inequalities, understanding the diversity and the complexities of participants (with ‘lived experience’), and experiential knowledge of service use through research is vital. An EoC perspective provides a more responsive alternative for involvement and a responsibility to consider interdependent aspects of lived realities. The responsibility of caring about ‘lived experiences’ extends to how categories are constructed, and associated processes of inclusion and exclusion. Without a critical approach to ‘lived experience’ as a category, experiences are homogenised within social structures that create inequalities. An EoC perspective asserts that ethical research, in the context of PPIE, also involves being competent within contexts that lack time, material resources with a demand for outcomes. Competency is the ability to question and interrogate ‘lived experience’ for ‘it is not individuals who have experiences, but subjects who are constituted through experience. Experience in this definition then becomes not the origin of our explanation, not the authoritative evidence that grounds what is known, but rather that which we seek to explain, that about which is produced’ [37, p. 25]. Being reflexive about ‘lived experience’ therefore shifts away from homogenising public partners and privileging ‘professional’ perspectives to, instead, a conversation about the complexities of knowledge production. This would be in keeping with the ideological underpinnings of PPIE to address epistemic injustice and research inclusivity.

The foregrounding of ‘lived experience’ as a way of addressing injustice stems from the explicit challenge posed by Black feminists to White feminists in the 1970s about women being equally oppressed [38]. Instead, Black feminists foreground Black women's ‘lived experience’ because of social constructs around race, reflected in an ideological hierarchical schema that positions them as inferior and leads to inequalities within the category of ‘woman’ [38]. Understanding ‘lived experience’, notably in health and social care research, Chikwira's [39] writings also stress intersectionality as significant, ‘to better understand how the different factors – including race/ethnicity, age, socio‐economic factors, gender, culture, religion, immigration status and class – *work together* in disadvantaging population groups and creating inequalities’ [39, emphasis mine]. Although a ‘rationale for intersectionality’ amongst professional stakeholders (which includes researchers) is accepted, questions are raised about the ‘the extent to which it is workable in practice’ [40, p. 3]. The barriers to adopting an intersectional approach stem, in part, from the understanding of categories, their production and the reproduction of social inequalities. A blanket notion of ‘lived experience’ dilutes the structural and political contexts within which health inequalities become established.

An intersectional approach, then, adds valuable insights for understanding the social determinants of health. In so doing, it enacts a form of competency, highlighted by the EoC perspective, which involves taking responsibility for moving beyond the simple adding of categories such as sexuality and disability, towards critically examining the processes of categorisation itself. Engaging with categorisations as socially constructed, which in turn are instrumental for shaping lived experiences, requires responsiveness to the power relations involved in knowledge production to tackle social injustice and health inequalities [37]. As such, an EoC approach demands reflexivity, challenging us to consider how identity categories are constructed and how they determine inclusion, representation and research relevance.

An intersectional analytical approach on the lived realities underlines that these are social and materially informed and do not occur in a vacuum, but rather within power relations that are historical and continue to reproduce inequalities [38]. Engaging with conceptual complexity is vital for inclusive PPIE. For example, ethnic minorities tend to be underrepresented, yet ethnicity as an aspect of ‘lived experiences’ is considered vital for recruitment into research studies (*and* PPIE activities) to be inclusive and for research relevancy. However, ethnicity and ‘race’ are often conflated or used interchangeably. For example, the NIHR Race Equality Framework [41] outlines consultations with ‘Black African‐, Asian‐ and Caribbean‐heritage communities’, conflating racialised groups with heritage with its core mission ‘for the ethnic diversity of people who are involved or participate in clinical research’ [41, p. 9], see [42]. ‘Race’ is a social construct, and there are no biological differences among human species. There is, nonetheless, an ideology behind ‘racial difference’ bolstered by discourses—that is, colonial and scientific racism—which has been used to subjugate and discriminate on account of biological determinism [43, 44, 45]. For example, categorisations around mental health are problematic as they reify masking racism and therefore ethnic identity translates into experiences of inequality [31]. Ethnicity is also a social construct and can be a way of understanding social groups by reference to dynamic and fluid cultural norms that foster a sense of identification and belonging [42]. Ethnicities, then, are made up of mutable and dynamic social and cultural practices such as language and are not bounded by space, time, physiology or genetics [42, 43]. Conflating ‘race’ with ethnicity enacts a sloppiness that removes the dynamism of ethnicity, which informs identities and practices contextually. There is a call to examine the use of ‘race’ and ethnicity in health to both distinguish and appropriately integrate race and ethnicity as social constructs impacting biological processes and health [42, p. 2]. Furthermore, if we disentangle ‘race’ from ethnicity, a space opens that allows for diversity within and amongst ethnic groups and is inclusive. For example, such a position underscores ‘whiteness’ as an ethnicity that displaces the normative conflation of race and culture as a feature of the ‘other’. An EoC perspective presents us with a commitment to discern knowledge production by recognising ‘whiteness’ as an ethnicity, rather than a neutral default, revealing how normative categories operate and reproduce inequalities [43].

Since its origin, PPIE has focused on patients and communities as recipients of services, providing a pathway for service improvement [33]. An EoC approach to PPIE would instead examine how (racialised) participants respond to the inherent ‘care’ they receive through their inclusion in PPIE. How marginalised participants respond to care‐receiving practices, such as PPIE, is addressed by Onwumere et al. [8], who highlight the ‘lived experiences’ of historical, institutional (including medical) and structural racism that have caused harm and fostered mistrust. They suggest that although mistrust is reflected upon and has led to an emphasis on gaining trust in PPIE guidelines, ‘the extent to which these activities and approaches are developed and refined using a racialised and/or intersectional approach and directly informed by the needs and lived experiences of homogeneous racialised communities are unclear’ [8, p. 3]. Onwumere et al. describe how being included as advisors, in research that pertains to their ‘lived experiences’, felt ‘novel’ yet uncomfortable for ‘Black Communities’ around ‘trustworthiness and different harms that they (and/or the Black communities that they identified with) might have endured as part of research initiatives’ [8, p. 4].

## 4 Conclusion

In conclusion, this paper draws attention to the ethical dimensions of PPIE, which is not only vital for ensuring the relevance and inclusivity of health research but also a crucial pathway for addressing health inequalities. Although PPIE activities often fall outside formal ethics review processes, they are nonetheless embedded within ethical considerations that shape relationships, responsibilities and outcomes. The paper proposes a cultural shift in the way PPIE, involving participants, considers ethical issues away from a procedural and technocratic perspective towards relational responsibility. An EoC perspective provides a constructive way to navigate these challenges, foregrounding empathy, attentiveness and relational responsibility as integral to ethical practice. By shifting the focus from procedural compliance and the narrow principle of ‘doing no harm’ towards the ongoing ethics of everyday engagement, this approach situates care at the centre of PPIE practice. Ultimately, adopting an EoC perspective encourages a more reflective, compassionate and accountable model of involvement that strengthens both the moral and methodological integrity of health research.

At its core, PPIE involves relational dynamics between researchers and participants across varying institutional settings. Adopting an EoC lens helps to ‘sensitise researchers to the complex moral responsibilities of their work in a highly hierarchical context and strengthen their reflexivity’ [24, p. 297]. Recognising that PPIE is embedded within power relations, the ethical focus must extend beyond the immediate research interaction to include the broader socio‐economic and political structures that shape lived experiences. An EoC approach, therefore, requires researchers to be competent in recognising how participants are positioned and conceptualised. It challenges static or individualised understandings of experience, calling instead for relational understandings that acknowledge difference and its intersections with systemic inequality. This paper has sought to critically examine the category of public and patient contributor, ‘participant’ and the concept of ‘lived experience’ through an intersectional approach, highlighting their social and political construction. In doing so, it offers a more nuanced and reflexive foundation for ethical practice in PPIE.

## Author Contributions

**Kanwal Mand:** conceptualisation, methodology, writing – original draft, writing – review and editing. **Anna Lavis:** conceptualisation, funding acquisition, writing – reviewing and editing. **Melissa Stepney:** conceptualisation, writing – reviewing and editing. **Roisin Mooney:** writing – reviewing and editing. **Kam Bhui:** funding acquisition, writing – reviewing and editing.

## Ethics Statement

This research received ethical approval from the STEM committee at the University of Birmingham. Approval ID ERN_1682‐Aug2024.

## Conflicts of Interest

The authors declare no conflicts of interest.

## Data Availability

Data sharing is not applicable to this article as no datasets were generated or analysed during the current study.
